# eFORGE v2.0: updated analysis of cell type-specific signal in epigenomic data

**DOI:** 10.1093/bioinformatics/btz456

**Published:** 2019-06-04

**Authors:** Charles E Breeze, Alex P Reynolds, Jenny van Dongen, Ian Dunham, John Lazar, Shane Neph, Jeff Vierstra, Guillaume Bourque, Andrew E Teschendorff, John A Stamatoyannopoulos, Stephan Beck

**Affiliations:** 1 Medical Genomics Group, UCL Cancer Institute, University College London, London WC1E 6BT, UK; 2 Altius Institute for Biomedical Sciences, Seattle, WA 98121, USA; 3 Department of Biological Psychology, Vrije Universiteit Amsterdam, Amsterdam 1081BT, The Netherlands; 4 European Molecular Biology Laboratory, European Bioinformatics Institute (EMBL-EBI), Cambridge CB10 1SD, UK; 5 Department of Human Genetics, McGill University and Génome Québec Innovation Center, Montréal H3A 0G1, Canada; 6 CAS Key Lab of Computational Biology, CAS-MPG Partner Institute for Computational Biology, Shanghai Institute for Biological Sciences, Chinese Academy of Sciences, Shanghai 200031, China; 7 Statistical Genomics Group, UCL Cancer Institute, University College London, London WC1E 6BT, UK

## Abstract

**Summary:**

The Illumina Infinium EPIC BeadChip is a new high-throughput array for DNA methylation analysis, extending the earlier 450k array by over 400 000 new sites. Previously, a method named eFORGE was developed to provide insights into cell type-specific and cell-composition effects for 450k data. Here, we present a significantly updated and improved version of eFORGE that can analyze both EPIC and 450k array data. New features include analysis of chromatin states, transcription factor motifs and DNase I footprints, providing tools for epigenome-wide association study interpretation and epigenome editing.

**Availability and implementation:**

eFORGE v2.0 is implemented as a web tool available from https://eforge.altiusinstitute.org and https://eforge-tf.altiusinstitute.org/.

**Supplementary information:**

Supplementary data are available at *Bioinformatics* online.

## 1 Introduction

DNA methylation (DNAm) is the main epigenetic mark assayed in the study of human diseases. The new EPIC BeadChip developed by Illumina can detect DNAm at over 850 000 genomic sites, extending the promoter-centric coverage of the 450k array to enhancers identified by the ENCODE and FANTOM5 projects (Andersson *et al.*, 2014; ENCODE Project Consortium, 2012), constituting a powerful and robust new tool for epigenome-wide association studies (EWAS) (Moran *et al.*, 2016).

The original version of eFORGE (Breeze *et al.*, 2016) employs multiple layers of epigenetic information, including data for open chromatin sites (DNase I hotspots) and histone marks (H3K4me1, H3K4me3, H3K27me3, H3K9me3 and H3K36me3) to detect cell types driving EWAS signal.

The updated version of eFORGE extends and enhances the tool, adding new features, such as simultaneous analysis across 15 chromatin states, detection of transcription factor (TF) motifs associated with EWAS signal, cumulative DNase I footprint analysis, EPIC array support and a browser to analyze TF occupancy in EWAS loci. Notably, we incorporate many of these features into a new web-based suite, eFORGE-TF, to aid the multilevel characterization of TF-associated EWAS mechanisms.

## 2 Description

**Fig. 1.**
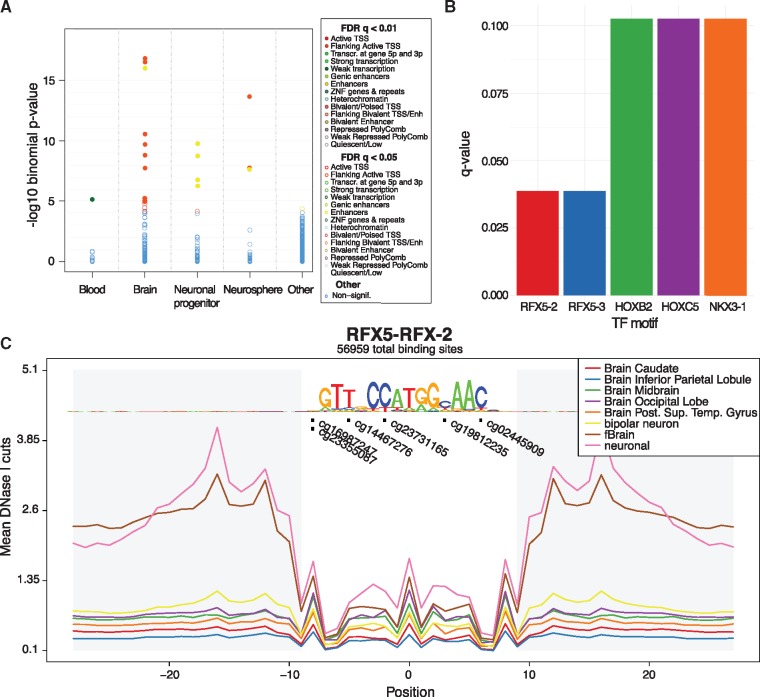
Output examples of eFORGE v2.0. (**A**) Enrichment in brain enhancers and TSS flanking chromatin states for the top 200 sites from an Illumina EPIC array study (Moran *et al.*, 2016). (**B**) TF motifs with the highest enrichment for the top 1000 study probes, including two RFX5 motifs. (**C**) Distribution of study sites in aggregated RFX5 footprints from brain samples shows 6 of the 1000 top study probes overlapping 5 different positions within the motif (*q*-value =0.038, hypergeometric test, BY correction)

eFORGE v2.0 is a software tool written in Perl and Python. eFORGE takes a list of EWAS array probes, and tests them for overlap enrichment with epigenetic tracks using an extensive database of 815 individual datasets. eFORGE currently includes tracks for DNaseI hotspots, 5 histone marks and 15 chromatin states. Probes can be filtered using a 1-kb proximity filter, and can be input in either BED or probe ID format. Statistical enrichment analysis is performed using a binomial test against an array-specific background. eFORGE outputs key information including Benjamini–Yekutieli-corrected *P*-values, sample IDs and lists of probes overlapping tracks in each sample. Both static and interactive charts and tables are provided to view results (Fig. 1A).

eFORGE chromatin state enrichment analysis extends previous eFORGE analyses by breaking down cell type-specific signal into regulatory element classes (e.g. subcategories of promoters, enhancers and transcribed regions). Furthermore, the activity of many of these classes is known to be associated with the binding of sequence-specific TFs. Interestingly, DNAm changes can also result as a consequence of the binding of sequence-specific TFs (Smith and Meissner, 2013).

To break down cell type-specific signal into particular TF groups we have generated eFORGE-TF, a software suite that allows for TF analysis at three levels. First, it analyses the EWAS probe list for TF motif enrichment across all known TF motifs (Fig. 1B). This analysis can optionally be conditioned to DNase I footprints. Second, eFORGE-TF performs a cumulative analysis across an aggregated set of DNase I footprints to visualize DNAm changes in relation to particular basepairs of an aggregated footprint (Fig. 1C). Third, eFORGE-TF provides a locus-specific gallery to inspect individual probes using a combination of TF motifs and DNase I footprints. These three analysis levels provide a robust and comprehensive assessment of TF associations for a given EWAS probe list.

In each gallery panel, eFORGE-TF includes an interactive browser for locus-specific analysis. This browser overlays multiple levels of information, such as footprints, TF motifs and genomic coordinates, providing an in-depth view into the TF associations of a particular locus. Such a tool can aid both EWAS interpretation and epigenome editing approaches, which can benefit from the characterization of local TF binding sites (Voigt and Reinberg, 2013).

More details, including an example eFORGE analysis, are available (Supplementary Material).

## 3 Conclusion

This updated and improved version of eFORGE constitutes an extended and powerful tool for the analysis of Illumina EPIC and 450k array data.

## Supplementary Material

btz456_Supplementary_Materials

## References

[btz456-B1] Andersson R. et al (2014) An atlas of active enhancers across human cell types and tissues. Nature, 507, 455–461. 24670763 10.1038/nature12787 PMC5215096

[btz456-B2] Breeze C.E. et al (2016) eFORGE: a tool for identifying cell type-specific signal in epigenomic data. Cell Rep., 17, 2137–2150. 27851974 10.1016/j.celrep.2016.10.059 PMC5120369

[btz456-B3] ENCODE Project Consortium (2012) An integrated encyclopedia of DNA elements in the human genome. Nature, 489, 57–74. 22955616 10.1038/nature11247 PMC3439153

[btz456-B4] Moran S. et al (2016) Validation of a DNA methylation microarray for 850, 000 CpG sites of the human genome enriched in enhancer sequences. Epigenomics, 8, 389–399. 26673039 10.2217/epi.15.114 PMC4864062

[btz456-B5] Smith Z.D., Meissner A. (2013) DNA methylation: roles in mammalian development. Nat. Rev. Genet., 14, 204–220. 23400093 10.1038/nrg3354

[btz456-B6] Voigt P., Reinberg D. (2013) Epigenome editing. Nat. Biotechnol., 31, 1097–1099. 24316647 10.1038/nbt.2756

