# Supplementary material for: eFORGE v2.0: updated analysis of cell type-specific signal in epigenomic data
Source: Bioinformatics. 2019 Jun 4;35(22):4767–9. doi: 10.1093/bioinformatics/btz456 (PMC6853678; doi:10.1093/bioinformatics/btz456)
Supplement: btz456_Supplementary_Materials [file btz456_supplementary_materials.docx]

Supplementary Materials for eFORGE v2.0: updated analysis of cell type-specific signal in epigenomic data

**Charles E Breeze^1,2,*^, Alex P Reynolds^2,*^, Jenny van Dongen^3^, Ian Dunham^4^, John Lazar^2^, Shane Neph^2^, Jeff Vierstra^2^, Guillaume Bourque^5^, Andrew E Teschendorff^6,7^, John A Stamatoyannopoulos^2^, Stephan Beck^1^**

1 Medical Genomics Group, Paul O’Gorman Building, UCL Cancer Institute, University College London, 72 Huntley Street, London WC1E 6BT, United Kingdom.

2 Altius Institute for Biomedical Sciences, 2211 Elliott Avenue 98121 Seattle

3 Department of Biological Psychology, Vrije Universiteit Amsterdam, 1081BT, Amsterdam, The Netherlands

4 European Molecular Biology Laboratory, European Bioinformatics Institute (EMBL-EBI), Wellcome Genome Campus, Hinxton, Cambridge, CB10 1SD, UK

5 Department of Human Genetics, McGill University, Montréal, Québec, Canada, McGill University and Génome Québec Innovation Center, Montréal, Québec, H3A 0G1, Canada.

6 CAS Key Lab of Computational Biology, CAS-MPG Partner Institute for Computational Biology, Shanghai Institute for Biological Sciences, Chinese Academy of Sciences, 320 Yue Yang Road, Shanghai 200031, China.

7 Statistical Genomics Group, UCL Cancer Institute, University College London, 72 Huntley Street, London WC1E 6BT, United Kingdom.

*Equal contribution

eFORGE is a recently updated web tool for analysis of cell type-specific signal in Illumina BeadChip data. The updated version of eFORGE provides multiple improvements compared to the previous version, adding analysis options such as EPIC array support, chromatin state enrichment analysis, TF motif analysis and DNase I footprint analysis. eFORGE v2.0 is thus the first version of eFORGE to include EPIC array support, though this addressing the needs of a growing sector of the EWAS field. In addition, eFORGE adds a new probe-centric browser to facilitate assessment of TF binding at a particular locus. In this document we demonstrate an example eFORGE analysis for a particular EPIC dataset.

In this example we focus on the analysis of data from an EPIC array study by Moran and others (Moran *et al.*, 2016). This study compared DNAm profiles between colon and neuronal cells, providing a set of DMPs that separate both tissues.

We obtained the study probe list from supplementary table 7, and sorted all probes by DNAm difference (“dif” column) in descending order. We then took the probeids from the top 200 probes for subsequent analysis.

Given that eFORGE only requires probe location information to perform subsequent analysis, it is important to highlight that only probeids were analysed beyond this point, and location for these probeids was automatically assigned by eFORGE and compared to location and annotation from 1000 EPIC array background probe sets. In a preliminary analysis, we used eFORGE to test for enrichment of the Moran et al. probes in DNase I hotspots from the Roadmap Epigenomics Consortium (2015 release), compared to 1000 background sets of EPIC array probes. Results show a strong enrichment for brain DNase I hotspots (figure 1).

**Figure** 1: eFORGE Consolidated Roadmap DHS results for top 200 probes from Moran et al. 2016. (eFORGE link: <https://eforge.altiusinstitute.org/files/0x5B90C4B25FAD11E888B1D3F654000C8C/Unnamed.850k.erc2-DHS.chart.pdf>).

We then performed similar DHS enrichment analyses across datasets from the ENCODE, BLUEPRINT and 2012 Epigenomics Roadmap datasets. Results across these consortia support the brain DNase I hotspot enrichment, with enrichments in brain and spinal cord. No enrichment is observed for any other tissue (figure 2).

**Figure** 2: eFORGE results for top 200 probes from Moran et al. 2016 across data from ENCODE (top), BLUEPRINT and 2012 Epigenomics Roadmap consortia (eFORGE links: ENCODE: <https://eforge.altiusinstitute.org/files/0xDA9ED6F45FAD11E884D880F754000C8C/Unnamed.850k.encode.chart.pdf>, BLUEPRINT: <https://eforge.altiusinstitute.org/files/0xEB7A16645FAD11E8BA92B6F754000C8C/Unnamed.850k.blueprint.chart.pdf>, 2012 Epigenomics Roadmap: <https://eforge.altiusinstitute.org/files/0xCDAB919E5FAD11E8A40F5BF754000C8C/Unnamed.850k.erc.chart.pdf>).

DHSs are markers for promoters, enhancers and other elements, constituting a general mark covering many cis regulatory element classes. To further dissect the underlying regulatory elements driving this brain signal we focused analysis on the 5 core histone marks mapped by the Epigenomics Roadmap consortium. Results reveal a brain H3K4me3 and H3K9me3 enrichment (figure 3). H3K4me3 is a mark enriched in active promoter elements and also present at active enhancers, and H3K9me3 is a mark enriched in heterochromatin and repressed regions.

**Figure** 3: eFORGE results for top 200 probes from Moran et al. 2016 across 5 histone mark datasets from the Epigenomics Roadmap consortium (including H3K4me1, H3K4me3, H3K27me3, H3K9me3 and H3K36me3, eFORGE link: <https://eforge.altiusinstitute.org/files/0xBA8B48CA5FAD11E8AE2545F754000C8C/Unnamed.850k.erc2-H3-all.chart.pdf>)

To further characterise the H3K4me3 and H3K9me3 enrichment revealed by eFORGE histone mark analysis we sought to perform an additional analysis across 15 chromatin states. eFORGE results show enrichments for "enhancer" and "flanking active TSS" categories for brain, neuronal progenitors and neurosphere (figure 4).

**Figure** 4: eFORGE results for top 200 probes from Moran et al. 2016 across 15 chromatin state datasets from the Epigenomics Roadmap consortium (eFORGE link: <https://eforge.altiusinstitute.org/files/0x55BB98285FAD11E88F9DC2F654000C8C/Unnamed.850k.erc2-chromatin15state-all.chart.pdf>)

We have thus analysed the top 200 probes from Moran et al., 2016 across data for DHSs, histone mark broadpeaks and chromatin states. We have designed the tool in such a way that further datasets can be added to local installations of the eFORGE web database. The code to generate the eFORGE web database is available from <https://github.com/charlesbreeze/eFORGE/blob/master/docs/eforge-db-construction/>. In addition, a graphical schematic of the eFORGE web sqlite database structure is available from <https://github.com/charlesbreeze/eFORGE/blob/eforge.v2.0/docs/eforge_2.0.web.db.schematic.svg>.

As in previous versions, eFORGE v2 includes an optional 1kb proximity filter to avoid biases associated with repeatedly testing proximal probes that present a strong DNA methylation correlation (Eckhardt *et al.*, 2006). For the 850k EPIC array, we have observed this filter removing up to 0.7% of probes in a random input set (7 out of 1000 probes, 1000 background tests), suggesting that eFORGE probe filtering avoids proximal probe bias in a typical input set without removing an excessive number of probes from analysis for the larger EPIC array.

In addition, eFORGE v2.0 currently contains 815 individual datasets (493 individual DHS datasets included the previous eFORGE publication, 195 individual histone mark datasets and 127 individual chromatin state datasets). Our online datatable includes all 815 individual datasets present in eFORGE (available at <https://docs.google.com/spreadsheets/d/1S1GCZmaPRXYHjFHCpY9XPWO_9NBlS0oi29uoq7weTbI/edit?usp=sharing>, column 1 indicates whether the datasets were added in eFORGE v2.0 or were added previously).

eFORGE v2 corresponds to developments in eFORGE web, which can also be set up for automated use in a cluster environment. For a simpler command line setup, eFORGE standalone remains in its original design for users to modify and experiment with new datasets.

Given that DNAm changes can result as a consequence of the binding of sequence-specific TFs, and that TFs are also involved in the formation of enhancers and other regulatory elements, we sought to identify TF motifs associated with the top 200 probes from Moran et al. 2016. We therefore performed an enrichment analysis across all 2256 TF motifs in eFORGE-TF. Results did not identify significant motifs at q<0.05 (figure 5). However, a p-value histogram shows evidence of anti-conservative p-values (figure 6), and we anticipated that testing a higher number of probes could reveal significant enrichments at q<0.05.


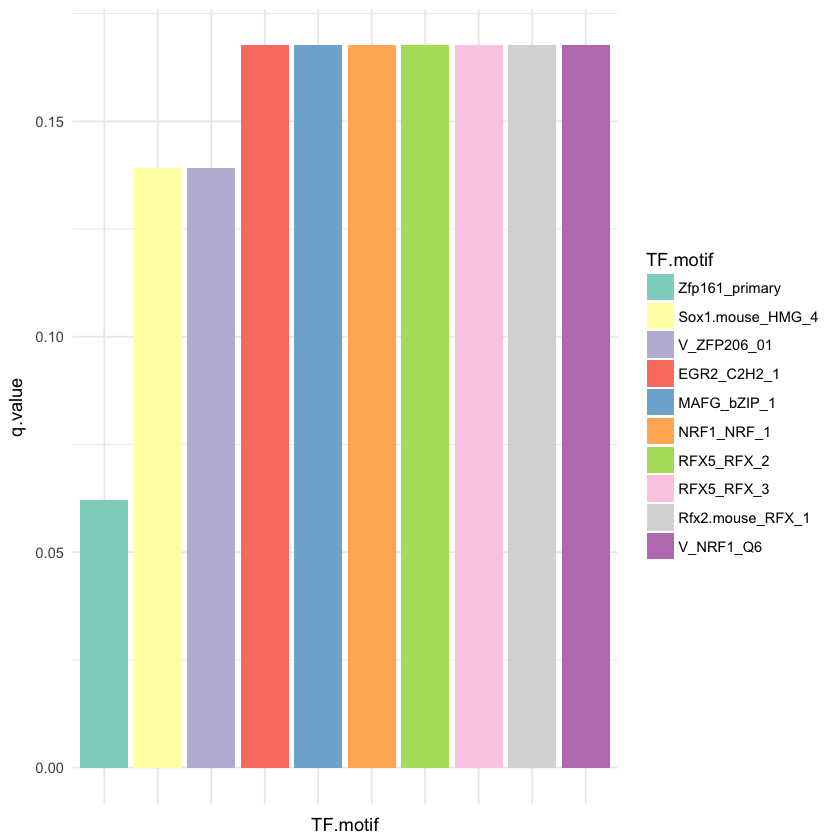


**Figure 5:** 10 most enriched TF motifs from eFORGE-TF analysis on the top 200 probes from Moran et al. 2016. No significant TF motifs are detected at q<0.05.


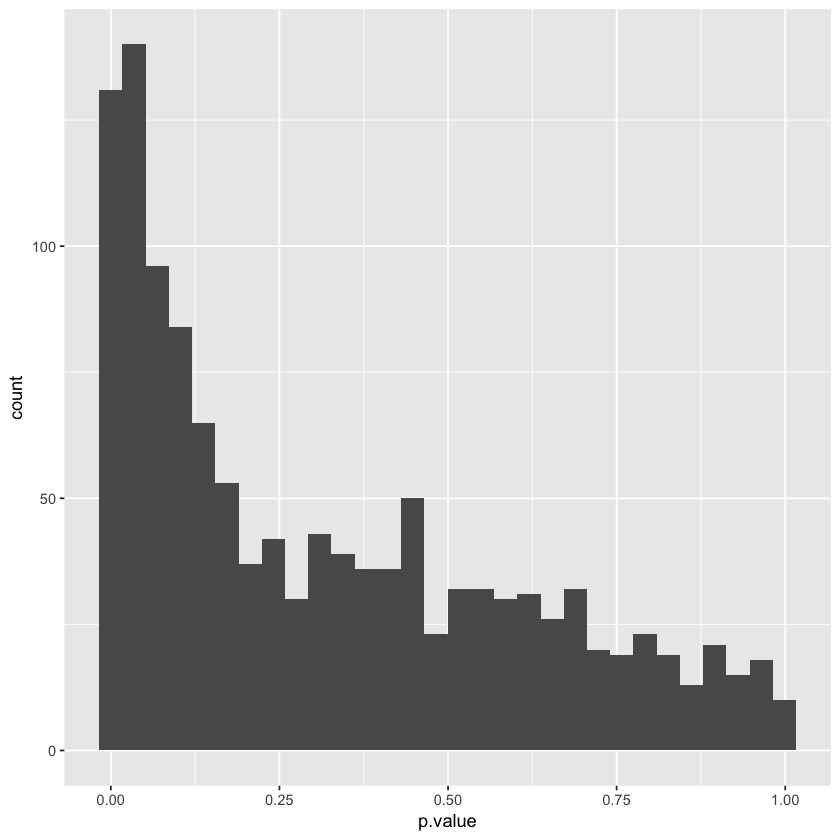


**Figure 6**: eFORGE-TF p-value histogram for top 200 probes from Moran et al. 2016 tested across 2256 TF motifs from the JASPAR, UniPROBE, TRANSFAC and Taipale DATABASES.

We therefore tested the top 1000 study probes (table 1) for enrichment analysis across 2256 TF motifs in eFORGE-TF. Results indicate a significant enrichment for motifs associated with transcription factor RFX5 (figure 7).


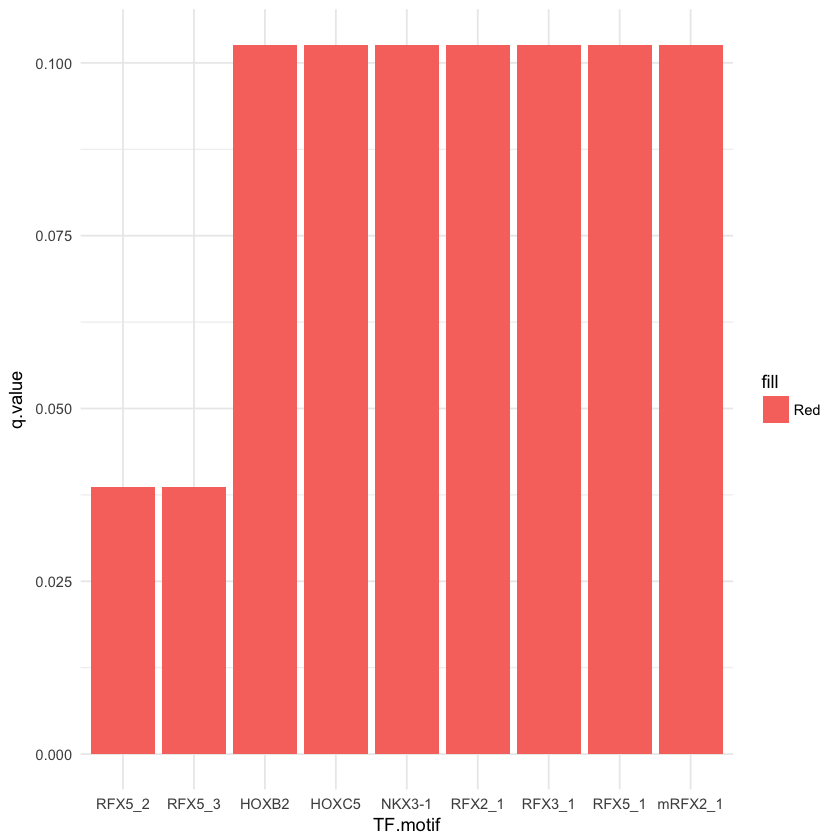


**Figure 7**: eFORGE-TF motif analysis reveals significant enrichment in two RFX5 motifs for top 1000 probes from Moran et al. 2016, and, in addition, low q-values for 4 additional motifs of TFs belonging to the RFX family.

eFORGE-TF analysis therefore points to RFX5 as a TF associated with several DMPs in this study. To better understand the role of DNAm changes at RFX5 sites we sought to characterise DMP location across the RFX5 footprint profile in brain. eFORGE-TF cumulative footprint analysis reveals that DMPs for the study by Moran et al. overlap several different positions covering most the RFX5 motif (figure 8).

**Figure 8:** Distribution of study sites and aggregated RFX5 footprints in brain. 6 of the 1000 top study probes overlap 5 different positions within the RFX5 motif. The RFX family is widely expressed in many tissues, including brain (Uhlén *et al.*, 2015) and has been associated with the regulation of genes linked to conditions such as dyslexia (Tammimies *et al.*, 2016) and bare lymphocyte syndrome (Mach *et al.*, 1996).

To improve our knowledge on the DMP-associated genes potentially regulated by RFX5 binding we sought to characterise RFX5 binding at specific study loci. An eFORGE-TF gallery was generated for RFX5 binding sites overlapping the top 1000 probes from Moran et al. (two of these sites are show in figure 9). Close examination of results suggests that RFX5 is likely to be bound at several of these loci, highlighting at least four binding sites likely to affect the differential methylation of CpG sites from this study.


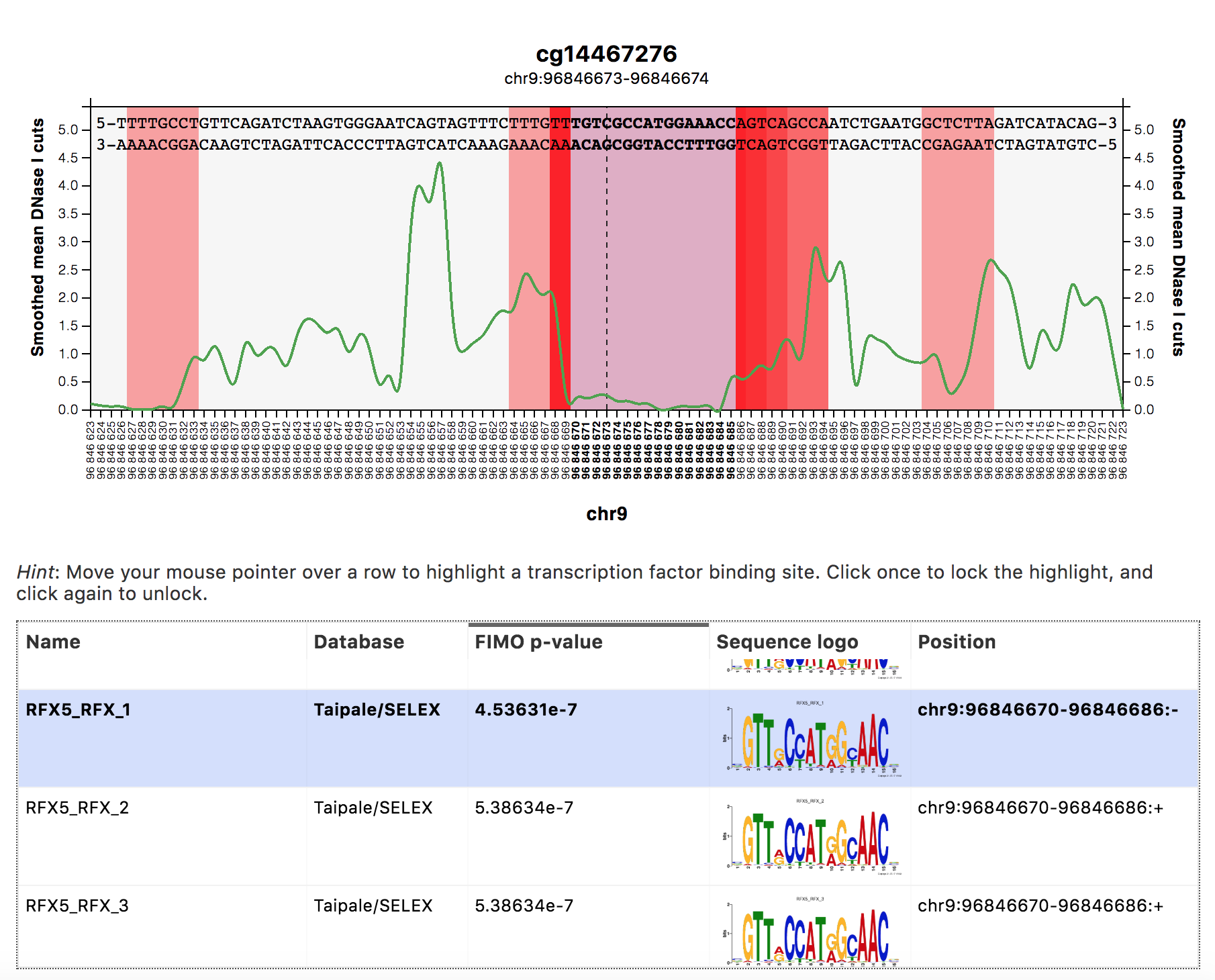


**
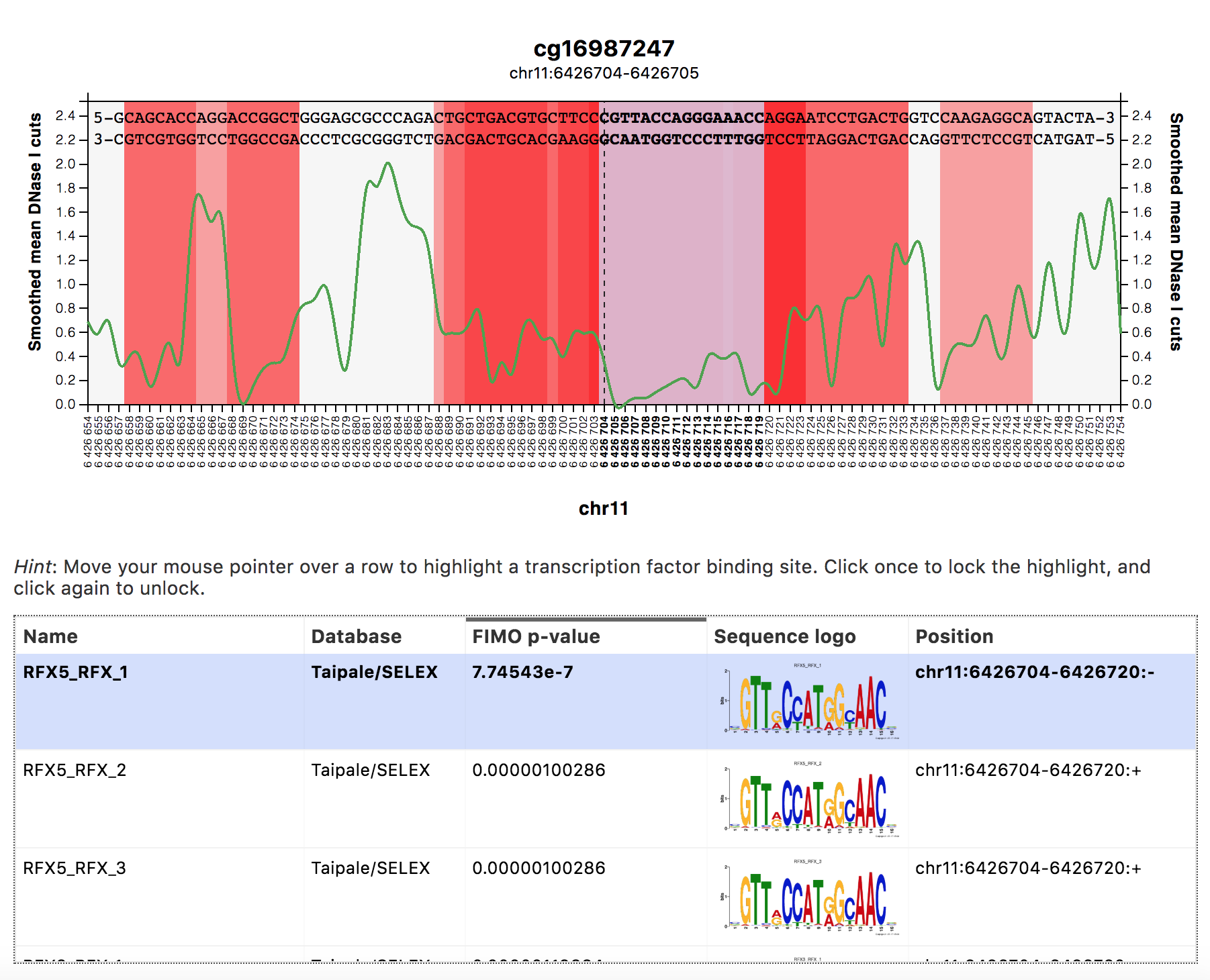
**

**Figure 9:** eFORGE-TF gallery examples for cg14467276 and cg16987247, two of the six probes from table 1 that overlap RFX5 motifs. [cg14467276, upper panel]: red regions indicate DNase I footprints, and the green line represents smoothed cutcount data. Probe location is indicated by a vertical dashed line in the centre of the graph. [cg14467276, lower panel]: TF motifs, including motifs for RFX5, are included in the lower panel. Given the interactive nature of the plot, hovering the cursor on a given motif in the lower panel will highlight its position on the graph in the upper panel. [cg16987247, upper panel]: same as for the upper panel of cg14467276. [cg16987247, lower panel]: same as for the lower panel of cg14467276.

We have thus used eFORGE-TF to characterise TF motifs and TF footprints associated with top probes from an EPIC array study by Moran et al. (2016). While eFORGE-TF currently only supports EPIC array analysis (<https://github.com/charlesbreeze/eFORGE-TF>), we intend to extend support for 450k arrays in the near future.

cg07823492

cg09787504

cg15760474

cg16490124

cg22664298

cg15365500

cg23298862

cg27628891

cg06688803

cg24525461

cg06032337

cg01248385

cg19113686

cg13144059

cg01589353

cg17008486

cg22029015

cg22346032

cg09010067

cg08407901

cg14114910

cg07178278

cg02138987

cg09684846

cg20843277

cg19683542

cg12798524

cg11172629

cg25308354

cg05893845

cg25124228

cg21913897

cg11246938

cg07637515

cg10124812

cg01400468

cg07864976

cg07209034

cg24471073

cg10787000

cg20595750

cg24080129

cg22686132

cg16581738

cg23231312

cg02192555

cg23938483

cg05403655

cg04780373

cg16083711

cg21023520

cg24542399

cg05833351

cg09307883

cg22789318

cg03673694

cg09998451

cg06866712

cg26407571

cg27494055

cg14447519

cg22155376

cg03796003

cg15183477

cg05003723

cg08093097

cg26511507

cg03485803

cg21017569

cg26634219

cg05409391

cg11630169

cg01111179

cg02035448

cg09618197

cg17671621

cg06164957

cg19658522

cg01620602

cg01018360

cg26889226

cg26799416

cg05636047

cg27088725

cg17510385

cg03449867

cg12655303

cg00792235

cg15084433

cg09137125

cg02413285

cg20536971

cg03339668

cg27317046

cg11920595

cg11265381

cg24105081

cg23401796

cg03025986

cg26705851

cg21526778

cg14122653

cg25258740

cg25411977

cg04129556

cg14870156

cg05402634

cg24948406

cg14142836

cg27456951

cg25415777

cg20244327

cg20782697

cg10677372

cg04904318

cg05788368

cg22300320

cg03326215

cg01245604

cg05274650

cg21457147

cg00102183

cg11281196

cg20659057

cg12908908

cg16995742

cg11830096

cg07592809

cg05937603

cg21233879

cg20342105

cg24897108

cg10027723

cg22727783

cg02013373

cg02208249

cg13102097

cg02914347

cg07437923

cg05093818

cg02024240

cg23401459

cg22660933

cg20546778

cg15733849

cg05599531

cg13102133

cg08045906

cg11423130

cg14218275

cg18208818

cg16953816

cg16452866

cg02389157

cg06683092

cg06791083

cg23546873

cg00084271

cg05422457

cg27587033

cg08869066

cg08557179

cg13075279

cg20036162

cg00366603

cg06783429

cg26693553

cg17454920

cg13246235

cg15913725

cg26567385

cg05426966

cg14846380

cg03967651

cg01963754

cg10772699

cg10197846

cg16987247

cg26097051

cg01610685

cg18528563

cg15443202

cg12468255

cg16360831

cg19079513

cg14467276

cg11085304

cg09052548

cg26190686

cg17619803

cg11068784

cg13396967

cg13951796

cg19256813

cg05583486

cg02676602

cg05845376

cg11452329

cg24812523

cg14203426

cg13450266

cg03494430

cg22169990

cg19072239

cg08069568

cg16516712

cg13711238

cg11051417

cg11811992

cg06084610

cg09762182

cg04860674

cg06293782

cg26880445

cg16299898

cg11677683

cg11551289

cg09119677

cg24605370

cg17243737

cg12363381

cg02902915

cg09102257

cg26276369

cg09363841

cg15624160

cg07596819

cg00054971

cg26382551

cg03540794

cg23934731

cg24250653

cg19453893

cg08850509

cg14667748

cg24799830

cg03688118

cg23882945

cg17135929

cg19277884

cg23843951

cg00035268

cg04477010

cg06646332

cg06662225

cg21935981

cg05679002

cg16683572

cg08522473

cg01141838

cg16490805

cg15775218

cg04942791

cg22262702

cg24594293

cg02994124

cg03522971

cg06413196

cg07691538

cg17415355

cg07015803

cg21529164

cg18074403

cg08200851

cg16450309

cg06257052

cg21193926

cg05931898

cg22615802

cg19021761

cg12556907

cg15621651

cg17058383

cg21585707

cg06590946

cg18173726

cg07809831

cg11452043

cg07795766

cg00009523

cg05235248

cg09625259

cg02176314

cg26354017

cg21567120

cg16659284

cg27397849

cg19812235

cg04263371

cg20229025

cg24214903

cg04429341

cg18232597

cg11476737

cg08908855

cg11511388

cg21332400

cg26952618

cg24697097

cg04868655

cg26904215

cg25264268

cg14923398

cg20370184

cg04337153

cg11155356

cg01512466

cg27123514

cg03972596

cg04404310

cg07574267

cg25537993

cg23731165

cg02721558

cg21546074

cg03729251

cg02622026

cg20168901

cg16777413

cg22573528

cg27437510

cg13802789

cg26607103

cg12503473

cg11005027

cg13913247

cg09918318

cg03463799

cg06766016

cg02643580

cg26750742

cg13710662

cg13235366

cg10888461

cg10648037

cg08569350

cg24121069

cg18579796

cg21104965

cg06981817

cg00868875

cg09053536

cg10989214

cg03828193

cg24145613

cg14072964

cg08050305

cg01708636

cg11002258

cg05111645

cg01442214

cg05697873

cg11716267

cg11264539

cg15720089

cg26728390

cg11059659

cg19592277

cg22659049

cg10127554

cg10273072

cg21033440

cg21398280

cg19514554

cg12829814

cg05771094

cg12280664

cg12975603

cg10418626

cg18167466

cg22598885

cg17627654

cg04110886

cg08931917

cg10159648

cg12136256

cg18054281

cg03170013

cg19278212

cg13300473

cg17504394

cg04503093

cg03959306

cg07450693

cg14470792

cg18459806

cg16964439

cg24064437

cg13125157

cg04336433

cg27277537

cg12608775

cg26814635

cg24325309

cg03001333

cg13983903

cg20435238

cg09741474

cg11254700

cg06059810

cg24453153

cg06844213

cg19684083

cg11503687

cg00081799

cg10052782

cg17614995

cg24715732

cg10359460

cg10436257

cg08264805

cg22741735

cg00746446

cg04459091

cg20090279

cg16034168

cg04985661

cg02972715

cg12852759

cg15474728

cg00870269

cg00728961

cg14755417

cg18064917

cg25735823

cg15488978

cg23460943

cg16290431

cg17914350

cg03145360

cg20679403

cg16634167

cg00660167

cg24601030

cg07947046

cg13433214

cg14588406

cg09647671

cg24303559

cg03061778

cg17553300

cg16784985

cg06962787

cg16915828

cg04497820

cg05347108

cg05432627

cg03736789

cg10070788

cg06834177

cg17681516

cg26319282

cg21775245

cg07355507

cg14869700

cg10137383

cg18041123

cg15286094

cg12976141

cg10559585

cg15373530

cg13407335

cg24128590

cg00713400

cg15199622

cg02260430

cg19126420

cg06588782

cg18828306

cg09581706

cg20181739

cg00528014

cg26075417

cg14096828

cg23355087

cg10412475

cg13387826

cg06084952

cg13508402

cg25895490

cg26470309

cg09145734

cg15669692

cg22078179

cg12905673

cg01983216

cg21203781

cg13909661

cg18456456

cg27508545

cg05617980

cg12209121

cg13916928

cg23479730

cg20540357

cg05876599

cg02896073

cg18301815

cg26867637

cg10397934

cg10495931

cg25616829

cg11824826

cg01851874

cg05037640

cg16986720

cg06964278

cg21095808

cg25705452

cg06623668

cg20125017

cg09377872

cg04147497

cg15593382

cg26118821

cg13856573

cg27623013

cg02445909

cg25297146

cg04807106

cg04080005

cg00053927

cg13246642

cg14636288

cg07479621

cg21887193

cg13871921

cg05686445

cg17831440

cg00905220

cg21609154

cg19612022

cg10536349

cg20061654

cg09761442

cg17865528

cg17621803

cg06790197

cg01323777

cg08851376

cg14893161

cg09414535

cg17778888

cg26275858

cg26757996

cg00675891

cg01577070

cg15839431

cg03502211

cg00576650

cg23548201

cg23013977

cg22235314

cg11239458

cg22686523

cg00866176

cg09065672

cg19423170

cg26739327

cg15161050

cg22865501

cg12462101

cg03544320

cg20379170

cg01753966

cg16140565

cg11222557

cg24018148

cg21617353

cg10620429

cg05820192

cg10556064

cg04206484

cg00820405

cg02403419

cg23573900

cg14549524

cg23778422

cg08861717

cg10907866

cg04747036

cg07672238

cg20691608

cg03738025

cg00491457

cg14500486

cg11580136

cg00017271

cg14411912

cg07055259

cg26364809

cg08854266

cg02292533

cg14642210

cg01156295

cg20202112

cg18792528

cg07102509

cg03667429

cg22350910

cg20419112

cg09850478

cg12354377

cg19855496

cg09650803

cg03966955

cg13447038

cg26831220

cg20296404

cg12314335

cg16047049

cg13888408

cg02324432

cg19856606

cg21973687

cg03724874

cg25422780

cg04695796

cg23055959

cg13040509

cg26912671

cg17744110

cg26891362

cg01052699

cg12501287

cg16822914

cg11019008

cg06607764

cg02920582

cg19205041

cg20082272

cg24213115

cg16239058

cg01636873

cg15411736

cg14163740

cg18473137

cg08256406

cg15645660

cg00164641

cg13603465

cg25920734

cg10414350

cg10054332

cg10670242

cg04118124

cg11712199

cg20583060

cg10140240

cg10331657

cg09317554

cg09417617

cg01578875

cg14957660

cg03959796

cg06966660

cg01287209

cg01101459

cg00025496

cg01736164

cg16026813

cg08718050

cg07366188

cg12988189

cg18934822

cg20026367

cg14684297

cg17253931

cg08753339

cg24093182

cg15428140

cg09750197

cg04074001

cg08041448

cg18753626

cg17380855

cg04315086

cg22782873

cg02032966

cg22352169

cg03257180

cg01277451

cg02382000

cg23251798

cg03048372

cg25127992

cg27467559

cg05438336

cg13258029

cg18384190

cg17864737

cg19003390

cg18590709

cg05598919

cg06133339

cg16252110

cg01876130

cg14476745

cg19841653

cg02520212

cg01089498

cg01246520

cg01414185

cg19999567

cg03318904

cg07266412

cg09990852

cg07336840

cg21022871

cg25061506

cg09894683

cg12642717

cg16676292

cg22148663

cg25981350

cg24053070

cg17803629

cg04266019

cg23184518

cg23528617

cg26443995

cg08439271

cg15831998

cg25023596

cg20602244

cg00037314

cg14159672

cg09280800

cg19199146

cg09559196

cg11274450

cg15622619

cg04346701

cg25342508

cg05830220

cg25762282

cg26440261

cg24368848

cg18611828

cg10577386

cg24185656

cg16639311

cg17538881

cg21879102

cg13143389

cg05753693

cg18390922

cg00531092

cg07780180

cg09096787

cg24145616

cg26539571

cg10585226

cg14957266

cg16669455

cg13514954

cg11158954

cg10979012

cg06345712

cg24335070

cg07517358

cg22381686

cg19220825

cg00701253

cg05940455

cg11571741

cg01091938

cg12952703

cg01895882

cg17738733

cg17758652

cg00655552

cg12512875

cg25920545

cg16217908

cg00897144

cg00912518

cg09631193

cg06881639

cg04494298

cg02518245

cg20920163

cg10014112

cg07821424

cg00197389

cg26528623

cg01985330

cg01292980

cg10778113

cg14051805

cg16230724

cg05660656

cg13943731

cg21211480

cg11479223

cg02580085

cg09306641

cg07907474

cg22614142

cg15287092

cg22202558

cg02640104

cg06027584

cg06484274

cg11280732

cg14608770

cg10566963

cg23144722

cg27207809

cg11879444

cg03141232

cg15647296

cg00552684

cg06060137

cg15461431

cg26537443

cg06228138

cg20267559

cg07934856

cg20983004

cg26363053

cg06239350

cg05293861

cg06484075

cg14344550

cg05190033

cg05203113

cg01875106

cg05191655

cg02090171

cg03078551

cg17572196

cg24875518

cg19080354

cg14962296

cg10019684

cg06478823

cg15188547

cg08384913

cg13957558

cg26209151

cg18556792

cg22457769

cg03190140

cg24953428

cg14033341

cg05014660

cg19391247

cg11571304

cg24413842

cg21223843

cg11622164

cg23920953

cg07985890

cg18175247

cg04208750

cg22478240

cg12050641

cg11953749

cg00466071

cg14282114

cg02852182

cg19789466

cg10077311

cg07340719

cg19780831

cg11827097

cg17071855

cg00287122

cg08564172

cg00644330

cg14084907

cg00807353

cg01288089

cg15237757

cg13500480

cg17394978

cg01201120

cg17148219

cg05826162

cg03661298

cg09328979

cg16327891

cg25557277

cg26711732

cg11855710

cg23676869

cg10236838

cg14425468

cg11576424

cg04694633

cg10187421

cg23516953

cg02821484

cg03336270

cg17445840

cg25621667

cg01622399

cg04844987

cg18811155

cg26305062

cg27102304

cg14480249

cg17252260

cg06711306

cg05907835

cg23837191

cg12441242

cg23005102

cg18174005

cg09225373

cg27628372

cg03617435

cg09948687

cg11619961

cg12190219

cg11125851

cg12258368

cg13084525

cg01155450

cg22515971

cg26477511

cg13679714

cg03639185

cg00994306

cg08731961

cg22854836

cg18448570

cg03857571

cg20893039

cg01948202

cg06221000

cg16469353

cg25504086

cg04048517

cg24982491

cg00589251

cg22797514

cg12832011

cg07754492

cg18680612

cg15179566

cg19651159

cg12577010

cg17759224

cg25485801

cg02890259

cg12729623

cg05205532

cg05702597

cg27599271

cg03092551

cg24945175

cg13404430

cg05192497

cg12846656

cg04446653

cg11036631

cg00438616

cg03532030

cg13126265

cg18434538

cg13492139

cg08249810

cg03396604

cg27032787

cg23889684

cg21801132

cg13884741

cg19100814

cg24028634

cg22416836

cg13756768

cg01355374

cg14810029

cg09551145

cg00924017

cg06267197

cg26959257

cg00700039

cg00695955

cg15258711

cg25543264

cg16933147

cg18443412

cg13573375

cg26758857

cg08469834

cg26266789

cg00698602

cg18577326

cg24380163

cg04331561

cg07713361

cg16271200

cg10078415

cg14882150

cg27064692

cg22396868

cg17825438

**Table 1**: top 1000 probes from Moran et al., Supplementary Table 7, when sorted in descending order by differential methylation (“dif” column). These probes (including the set of top 200 probes in this table) were used as input in the eFORGE v2.0 analyses presented in this document.

**References**

Eckhardt,F. *et al.* (2006) DNA methylation profiling of human chromosomes 6, 20 and 22. *Nat. Genet.*, **38**, 1378–1385.

Mach,B. *et al.* (1996) Regulation of MHC class II genes: lessons from a disease. *Annu. Rev. Immunol.*, **14**, 301–331.

Moran,S. *et al.* (2016) Validation of a DNA methylation microarray for 850,000 CpG sites of the human genome enriched in enhancer sequences. *Epigenomics*, **8**, 389–399.

Tammimies,K. *et al.* (2016) Ciliary dyslexia candidate genes DYX1C1 and DCDC2 are regulated by Regulatory Factor X (RFX) transcription factors through X-box promoter motifs. *FASEB J.*, **30**, 3578–3587.

Uhlén,M. *et al.* (2015) Tissue-based map of the human proteome. *Science*, **347**, 1260419.
